# PEX11β induces peroxisomal gene expression and alters peroxisome number during early *Xenopus laevis *development

**DOI:** 10.1186/1471-213X-11-24

**Published:** 2011-04-28

**Authors:** Mark A Fox, Logan A Walsh, Michelle Nieuwesteeg, Sashko Damjanovski

**Affiliations:** 1 Department of Biology, University of Western Ontario, 3053 Biological and Geological Sciences Building, 1151 Richmond Street North, London, ON, N6A 5B7, Canada

## Abstract

**Background:**

Peroxisomes are organelles whose roles in fatty acid metabolism and reactive oxygen species elimination have contributed much attention in understanding their origin and biogenesis. Many studies have shown that *de novo *peroxisome biogenesis is an important regulatory process, while yeast studies suggest that total peroxisome numbers are in part regulated by proteins such as Pex11, which can facilitate the division of existing peroxisomes. Although *de novo *biogenesis and divisions are likely important mechanisms, the regulation of peroxisome numbers during embryonic development is poorly understood. Peroxisome number and function are particularly crucial in oviparous animals such as frogs where large embryonic yolk and fatty acid stores must be quickly metabolized, and resulting reactive oxygen species eliminated. Here we elucidate the role of Pex11β in regulating peroxisomal gene expression and number in *Xenopus laevis *embryogenesis.

**Results:**

Microinjecting haemagglutinin (HA) tagged Pex11β in early embryos resulted in increased RNA levels for peroxisome related genes PMP70 and catalase at developmental stages 10 and 20, versus uninjected embryos. Catalase and PMP70 proteins were found in punctate structures at stage 20 in control embryos, whereas the injection of ectopic HA-Pex11β induced their earlier localization in punctate structures at stage 10. Furthermore, the peroxisomal marker GFP-SKL, which was found localized as peroxisome-like structures at stage 20, was similarly found at stage 10 when co-microinjected with HA-Pex11β.

**Conclusions:**

Overexpressed Pex11β altered peroxisomal gene levels and induced the early formation of peroxisomes-like structures during development, both of which demonstrate that Pex11β may be a key regulator of peroxisome number in early Xenopus embryos.

## Background

Peroxisomes are single-membrane bound organelles found ubiquitously in eukaryotic cells. They house more than 50 matrix enzymes that participate in a diverse array of metabolic processes including the β-oxidation of very long chain fatty acids (VLCFA) and α-oxidation of long branched-chain fatty acids [1]. Peroxisomes also contain oxidases that produce the corrosive byproduct hydrogen peroxide (H_2_O_2_) [2]. H_2_O_2 _and other dangerous reactive oxygen species (ROS) are then converted to innocuous products such as water and molecular oxygen by catalase and other enzymes within the peroxisome and in other cellular compartments [3]. Because of their complex roles in both cellular metabolism and ROS elimination, peroxisome function is strongly related to cellular development and eventual cellular senescence when their functionality begins to fail.

While cellular aging and senescence are well characterized by peroxisomal dysfunction [4], little is known about the origin of these organelles, particularly during embryonic development. Important players in the regulation of overall peroxisome numbers are the peroxisome proliferator-activated receptors (PPARs), which were first identified in the early 90s in mice [5]. Three types of PPARs have been identified (alpha, gamma, and delta) that function as transcription factors and play critical physiological roles as lipid sensors and regulators of lipid metabolism, as well in the regulation peroxisome numbers [6]. Total peroxisome numbers, and as importantly, peroxisome biogenesis, involves the production of proteins termed peroxins; nuclear encoded by Pex genes, synthesized on free polyribosomes in the cytosol and post-translationally transported into the peroxisomal matrix and membrane [7]. Peroxins can facilitate peroxisomal membrane function, biogenesis and division, and the transport of specific cytosolic proteins into the peroxisomal matrix via one of two peroxisomal targeting signals (PTS) [8]. The PTS2 signal sequence is a complex amino terminal signal composed of N/K-L-X5-Q-H/L, while the PTS1 consists of the C-terminal amino acid sequence SKL and a conserved variant form, KANL [9]. Studies have shown proteins with the SKL signal have a higher affinity for peroxisomes than proteins with the relatively weaker KANL signal [10]. In addition to providing functionality within peroxisomes, these cytoplasm-to-peroxisome protein import pathways have been proposed as a necessary mechanism to increase peroxisome numbers from existing peroxisomes [11]. While peroxisome number may be augmented though signal transduction [12,13], the total number of peroxisomes in a cell is regulated by; (i) peroxisome *de novo *biogenesis, (ii) peroxisome proliferation by division and (iii) peroxisome degradation by pexophagy, an autophagy-related process [14].

Multiple studies on Pex11 proteins have contributed to understanding their role in peroxisome division, although the specific molecular mechanisms that regulate their function are poorly understood [13]. Expression levels of Pex11 peroxins are directly correlated with peroxisome numbers [15]. For example, a Pex11p knock-down in yeast significantly reduced the amount of cellular peroxisomes, whereas, Pex11p overexpression increase their numbers [16]. A similar ability to promote peroxisome proliferation was also reported in humans [13], rodents and protozoan models [17]. All of these studies support a direct role for the Pex11-family in peroxisome division *in vitro*, though little is known about their role during embryogenesis.

It is unknown whether peroxisomes exist in fertilized eggs, or in early stage vertebrate embryos. While early frog development requires glycogen and lipid reserves to be oxidized, and protein and yolk reserves to be metabolized, surprisingly little is known about the regulation of yolk, vitellogenin and lipid metabolism in oviparous animals such as frogs. Early histological staining studies revealed that yolk and lipid utilization follow gastrulation, but preceded cell differentiation. Selman and Pawsey revealed that frog yolk and lipid utilization took place ventral to the archenteron just prior to stage 20, and within the developing myotomes by stage 23 [18]. Other histochemical studies have also shown that yolk and lipid metabolism occurs within the somites as they begin to differentiate between stages 17-24 [19]. Yolk is then metabolized in most differentiating tissues in the embryo after stage 30 [19]. This tissue specific utilization of yolk has been more recently confirmed using a variety of approaches including the examination of pH changes, and the involvement of proteases such as cathepsin D, and inhibitors such as EP45/pNiXa/Seryp [20-23]. This tissue specific regulation of yolk metabolism during embryogenesis suggests complex underlying developmental controls of these processes.

While peroxisomes are needed for metabolism and ROS regulation, their origins and biogenesis within the embryo are poorly understood. Here we examine the level of expression of peroxisomal genes Pex1, Pex3, Pex5, Pex11β, catalase and PMP70, as well as PPARα, δ, and γ in a *X. laevis *cell line, and during embryonic development. We test the hypothesis that Pex11β has the ability to induce peroxisomal gene expression *in vitro*, and induce early increase in peroxisome number *in vivo*. Our results demonstrate that overexpression of Pex11β can increase the number of peroxisomes in Xenopus A6 cells *in vitro*, and induce an early-onset to peroxisome-like structures during Xenopus embryogenesis *in vivo*. We propose that Pex11β plays a direct role in peroxisome divisions, and additionally, regulating the timing of peroxisome biogenesis during *X. laevis *embryonic development.

## Results

### Pex11β altered the RNA levels of peroxisome related genes in *X. laevis *A6 cells

**Figure 1:**
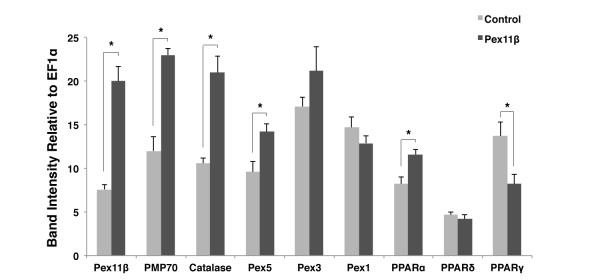
**Overexpressing HA-Pex11β altered peroxisome related gene expression in Xenopus A6 cells**. RT-PCR analysis of peroxisomal genes was performed before and after transfection of A6 cells with HA-Pex11β. Two days following transfection 250 ng of reverse-transcribed A6 cell RNAs from control and treatment samples (n = 3) were subject to PCR amplification using specific primers for the peroxisome related genes; Pex11β, PMP70, catalase, Pex5, Pex3, Pex1, PPARα, -δ, and -γ. The respective mRNA levels represent measures of mid-log phase RT-PCR product band intensities, relative to levels of EF1α. Genes whose levels were altered significantly, as assessed by an independent samples t-test, are denoted with an asterisk. Pex11β, PMP70, catalase, Pex5 and PPARα displayed elevated levels of expression following treatment, while PPARγ displayed reduced expression. *P < 0.05*, n = 3. Values presented are the means ± SE.

We first investigated if Pex11β could alter the RNA levels of the peroxisome related genes Pex1, Pex3, Pex5, Pex11β, catalase, PMP70, PPARα, -δ, and -γ in *X. laevis *A6 kidney epithelial cells, something that has not been demonstrated in Xenopus before. A6 cells were transfected with plasmids designed to express Xenopus HA-Pex11β, or control full-length GFP. Semi-quantitative RT-PCR analyses revealed a significant increase in Pex11β, PMP70, catalase, Pex5 and PPARα, but a significant decrease in PPARγ mRNA levels, following HA-Pex11β overexpression in A6 cells (Figure 1). No significant changes were found in levels of Pex3, Pex1, nor in PPAR δ mRNA (Figure 1).

### Pex11β increased hallmark peroxisomal protein levels in *X. laevis *A6 cells

**Figure 2:**
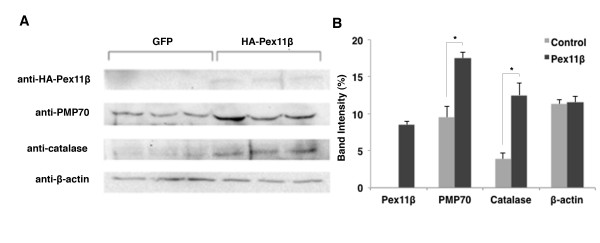
**Overexpression of HA-Pex11β in A6 cells increased catalase and PMP70 protein levels**. Western blotting revealed elevated proteins levels of catalase and PMP70 following the transfections of HA-Pex11β, but not GFP, in three samples of A6 cells (A). An HA antibody confirmed the translation and presence of HA-Pex11β in transfected cells (right 3 lanes) versus GFP transfected control cells (left 3 lanes). Catalase and PMP70 antibodies also displayed altered band intensities of each respective protein in HA-Pex11β transfected cells (right 3 lanes) versus GFP transfected cells (left 3 lanes). Protein loading in each lane was confirmed via a β-actin antibody. The Western blot signals were digitized and data were quantified and analyzed to statistically compare protein levels (B). There was a significant increase in the levels of catalase and PMP70 following overexpression of Pex11β, while there was no difference in the levels of β-actin. Statistical relevance of discrepancies between groups (asterisks) was assessed by an independent samples t-test. *P < 0.05*, n = 3. Values presented are the means ± SE.

Since overexpression of HA-Pex11β increased the mRNA levels of catalase and PMP70, we next wanted to determine if there were actual increases in the protein levels of these hallmark peroxisomal proteins. A6 cells were transfected as previously described and protein samples were isolated for Western blots. Using a specific HA antibody, Western blot analysis confirmed bands of expected sizes for HA-Pex11β (63 kDa) in transfected samples (Figuer 2A), confirming the integrity of the HA-tagged construct. Western blot analyses with catalase and PMP70 specific antibodies also revealed bands of expected sizes for both PMP70 (70 kDa) and catalase (55 kDa) (Figure 2). A significant increase in catalase and in PMP70 were found following HA-Pex11β overexpression versus control untransfected samples (Figure 2A, left three GFP lanes versus right three HA-Pex11β lanes, and quantified in Figure 2B). The use of anti-β-actin demonstrated the relative protein levels in each lane.

### Overexpression of Pex11β increased peroxisome numbers in *X. laevis *A6 cells

**Figure 3:**
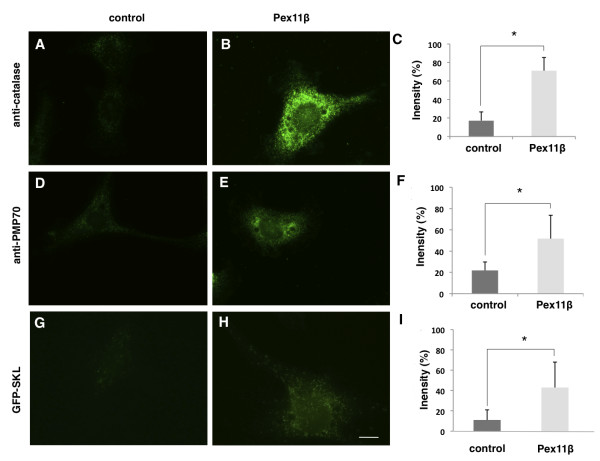
**Overexpression of HA-Pex11β in A6 cells increased peroxisome numbers**. A, D, G are untransfected cells, while B, E, H have been transfected with HA-Pex11β. G and H are additionally transfected with GFP-SKL. Using identical imaging and photography parameters, indirect immunofluorescence using a catalase antibody revealed lower levels of immunofluorescence in untransfected cells (A) versus transfected (B) cells. Similarly, indirect immunofluorescence using a PMP70 antibody revealed lower levels of signal in untransfected cells (D) versus transfected (E). Direct fluorescence for GFP revealed a diffuse signal from GFP in HA-Pex11β untransfected cells, (G) versus the presence of punctate structures in HA-Pex11β transfected cells (H). All images were captured using identical fluorescent settings. The relative fluorescence intensity in 10 regions of twenty randomly imaged cells was quantified using Northern Eclipse software. Graphs on the right represent the average fluorescence intensity of untransfected versus HA-Pex11β transfected cells. Values presented are the means ± SE. Significance at *P < 0.05 *was determined using Student's t-test, n = 25.

Although recent studies in several eukaryotic cell lines have revealed that Pex11-proteins can independently increase peroxisome-like structures [18], we tested the hypothesis that Pex11β could induce an early onset to peroxisome biogenesis during Xenopus embryogenesis. However, we first tested whether overexpression of Pex11β could increase peroxisome-like structures and peroxisome number in *X. laevis *A6 cells. Two days following transfection of HA-Pex11β, cells were fixed for immunocytochemistry and probed with PMP70 and catalase antibodies. This allowed us to examine the distribution of PMP70 and catalase protein, and also to quantify their relative protein levels using a fluorescent secondary antibody. Our results indicated that overexpression of HA-Pex11β significantly increased the amounts of both catalase levels (Figure 3A versus 3B) and PMP70 levels (Figure 3D versus 3E) versus control, as determined by quantifying the relative levels of indirect fluorescent; catalase indirect fluorescence levels increased >3 fold (Figure 3C), while PMP70 indirect fluorescence levels increased >2.5 fold (Figure 3F).

As the increased levels of PMP70 and catalase fluorescent signals may not specifically be related to peroxisome function, we next tested if overexpression of HA-Pex11β could also increase the number of peroxisomes using the peroxisomal marker GFP-SKL as a detection assay. This PTS1 tagged GFP will localize to punctate-like structures in the cytosol when imported into peroxisomes. A6 cells were co-transfected with HA-Pex11β and GFP-SKL, or GFP-SKL alone. Two days following transfection, peroxisome-like structures were assessed by direct immunofluorescence. Our results showed a significant increase in the number GFP-containing bodies (>2-fold, Figure 3I) in cells that expressed HA-Pex11β versus cells transfected with GFP-SKL alone (compare Figure 3G and 3H).

### Pex11β increased peroxisome related gene expression during *X. laevis *embryogenesis

**Figure 4:**
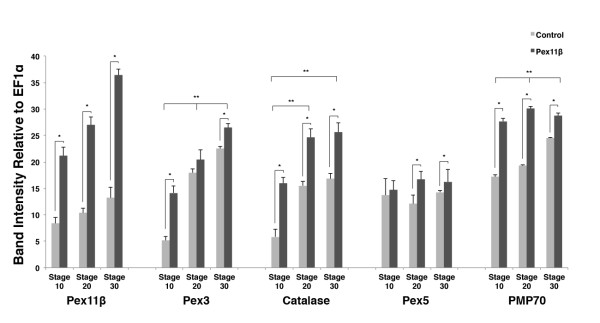
**Embryonic overexpression of HA-Pex11β elevated Pex3, catalase and PMP70 levels**. The respective mRNA levels represent measures of mid-log phase RT-PCR product band intensities, relative to levels of EF1α. RT-PCR analysis during normal embryogenesis revealed that the levels of all genes examined, with the exception of Pex5, increased as development progressed. First, repeated measures ANOVAs were carried out entering all RNA levels at all 3 stages. When significant, paired samples t-tests were carried out between stage levels in control embryos. This would reveal significant changes in RNA levels of the genes examined during normal development. Significant changes in RNA levels of a gene between stages is represented by the double asterisk ** (*P < 0.05*). Expression increases with development and there are differences in the levels of Pex3 and PMP70 between all stages, 10vs20, 20vs30 and 10 versus 30. For catalase there are differences between stages 10vs20 and 10vs30 but NOT between 20 versus 30. There are no significant differences in RNA levels between the tested developmental stages for Pex11β or Pex5. As the means were correlated, MANOVAs were carried out on the mean RNA levels of the treatment and control groups at each stage for each gene. All MANOVAs displayed significant effects of condition (Wilks lambda, *P < 0.05*) and so univariate ANOVAs were carried out. Treatments that resulted in significantly higher levels of gene expression following treatment are represented by a single asterisk. Pex11β, catalase and PMP70 all displayed significant increases in RNA levels all stage 10, stage 20 and stage 30 following treatment. Pex3 displayed elevated expression at only stage 10 and 30, while Pex5 displayed differences only at stages 20 and 30. (*Ps < 0.05*). n = 3. Values presented are the means ± SE.

We next examined the effects of increased Pex11β *in vivo*, by investigating changes in expression of specific peroxisomal genes, following the microinjection HA-Pex11β RNA into early Xenopus embryos. To establish a foundation, the temporal expression of five peroxisomal genes was first analyzed during the developmental stages of gastrulation (stage 10), neural tube closure (stages 20) and organogenesis (stage 30). In general, with the exception of Pex5, all peroxisomal genes examined in control embryos increased in expression as development progressed, with their lowest expression levels at stage 10, and highest at stage 30 (Figure 4, significance between stages denoted by double asterisks). Pex5 expression in control embryos does not alter significantly between stages 10 and 30. The increase in PMP70 RNA level between stages 10 and 20 differs from that a previously described decreasing trend between stages 12 and 20 [23]. This difference could be due to the examination of different stages (stage 10 vs 12), though in both cases the more important trend of increasing levels towards stage 30 and beyond is the same. Following microinjection of HA-Pex11β RNA there were significantly increased RNA levels of catalase and PMP70 *at *stages 10, 20 and 30 (Figure 4, single asterisk) versus control RNA levels of expression for each gene. Microinjecting HA-Pex11β also resulted in significant increases of Pex3 *at *stages 10 and 30, as well as Pex5 *at *stages 20 and 30. There were no significant changes in Pex3 at stage 20, nor Pex5 *at *stage 10. Changes in Pex11β levels following microinjection of HA-Pex11β reflect the presence of the HA-Pex11β construct.

**Figure 5:**
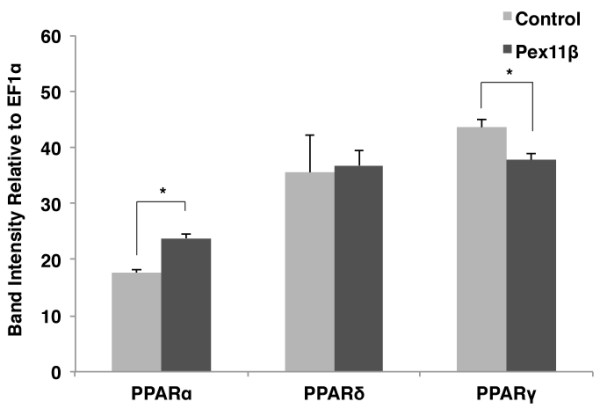
**Overexpression of HA-Pex11β did altered PPARα and γ, but not δ, gene expression during early *X. laevis *embryogenesis**. The respective mRNA levels represent measures of mid-log phase RT-PCR product band intensities, relative to levels of EF1α. Using a similar approach and analysis as used in Figure 4, RT-PCR analysis of RNA isolated from control stage 10 embryos and HA-Pex11β injected embryos revealed significant changes in the expression of PPARα, and PPARγ, but not PPARδ. PPARα levels were elevated by treatment, while PPARγ levels were reduced. *P < 0.05*, n = 3. Values presented are the means ± SE.

Further, as ectopic Pex11β significantly decreased PPARγ RNA levels in A6 cells, we investigated whether ectopic Pex11β would similarly alter PPAR levels within embryos. The injection of Pex11β into embryos significantly increased levels of PPARα, significantly decreased levels of PPARγ, but did not change levels of PPARδ RNA (Figure 5), a pattern similar to that seen in the A6 cells.

### Catalase and PMP70 antibodies reveal early punctate organelle structures during *X. laevis *embryogenesis following Pex11β injections

**Figure 6:**
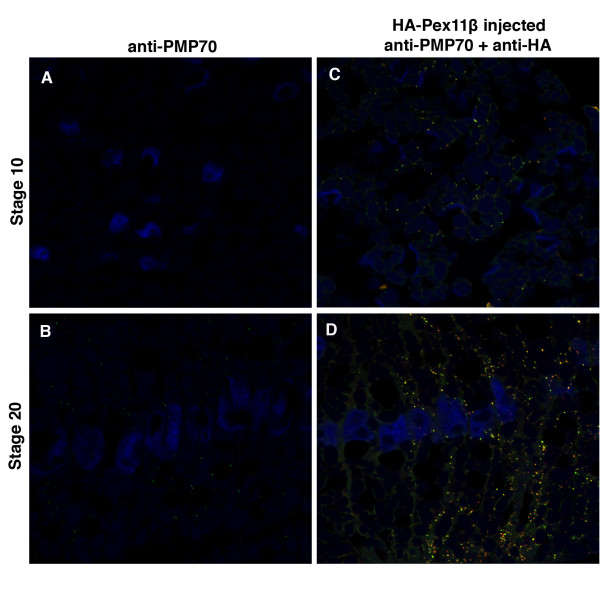
**Microinjecting HA-Pex11β RNA increased PMP70 immunofluorescence levels during *X. laevis *embryogenesis**. Both control (A and B) and HA-Pex11β injected (C and D) embryos at developmental stages 10 (A and C) and 20 (B and D), were fixed then sectioned for immunohistochemical analysis in somites for PMP70. At stage 10 PMP70 protein is undetected in somitic mesoderm under control conditions (A), whereas following microinjection of HA-Pex11β PMP70 protein is detectable in punctate structures (C). At stage 20, PMP70 protein was detected in both control and following microinjecting HA-Pex11β (B and D). An HA antibody was also used to confirm the ectopic presence of HA-Pex11β. DAPI (blue), PMP70 (green), HA-Pex11β (red), colocalization of HA-Pex11β and catalase (yellow). Images were taken at 60× magnification.

**Figure 7:**
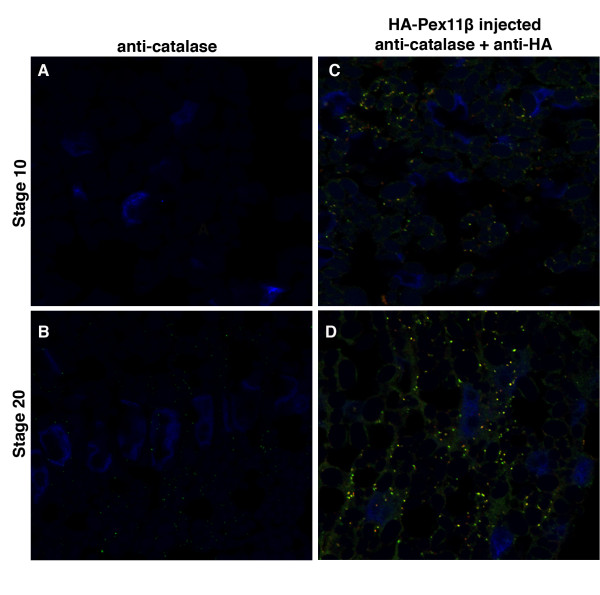
**Microinjecting HA-Pex11β RNA increased catalase immunofluorescence levels during *X. laevis *embryogenesis**. Both control (A and B) and HA-Pex11β injected (C and D) embryos at developmental stages 10 (A and C) and 20 (B and D), were fixed then sectioned for immunohistochemical analysis in somites for catalase. At stage 10 catalase protein is undetected in somitic mesoderm under control conditions (A), whereas following microinjection of HA-Pex11β catalase protein is detectable in punctate structures (C). At stage 20, PMP70 protein was detected in both control and following microinjecting HA-Pex11β (B and D). An HA antibody was also used to confirm the ectopic presence of HA-Pex11β. DAPI (blue), PMP70 (green), HA-Pex11β (red), colocalization of HA-Pex11β and PMP70 (yellow). Images were taken at 60× magnification.

To determine if Pex11β could induce an early onset to peroxisome-like structures during Xenopus development, we used immunohistochemistry to visualize changes in the embryonic distribution of PMP70 and catalase, in response to microinjecting HA-Pex11β RNA. Fertilized embryos were microinjected with HA-Pex11β RNA, fixed at stages 10 and 20, and sectioned for immunohistochemistry. PMP70 and catalase signals were undetected in stage 10 under control conditions (Figure 6A and 7A) using specific antibodies. Punctate structures were visualized within the somites at stage 20 in control sections, using PMP70 (Figure 6B), and catalase (Figure 7B) specific antibodies. Following microinjection of HA-Pex11β, we were able to detected punctate PMP70 (Figure 6C) and catalase (Figure 7C) signals at stage 10 in pre-somitic mesoderm, and increased levels of immunofluorescence for both proteins in stage 20 somites compared to control uninjected embryos (compare Figure 6B vs 6D, and 7B vs 7D).

### Overexpression of Pex11β triggered an early-onset to peroxisome accumulation during Xenopus embryogenesis

**Figure 8:**
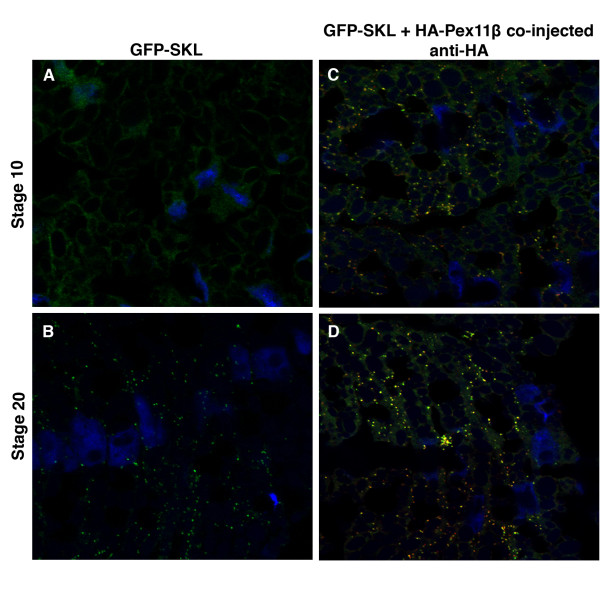
**Microinjecting HA-Pex11β RNA increased the number of peroxisome-like GFP-SKL structures during *X. laevis *embryogenesis**. Both control (A and B) and HA-Pex11β injected (C and D) embryos at developmental stages 10 (A and C) and 20 (B and D), were fixed then sectioned for immunohistochemical analysis in somites for GFP-SKL. At stage 10, GFP fluorescence is present as a faint diffuse stain under control conditions (A), whereas following microinjection of HA-Pex11β punctate GFP structures could be seen at this early stage (C). At stage 20, punctate GFP structures were detected in both control and following microinjecting HA-Pex11β, where numbers were increased in the injected samples. An HA antibody was also used to confirm the ectopic presence of HA-Pex11β. DAPI (blue), GFP (green), HA-Pex11β (red), colocalization of HA-Pex11β and GFP-SKL (yellow). Images were taken at 60× magnification.

In order to determine when peroxisomes are first present during embryonic development, focusing on the dorsal mesoderm, we microinjected GFP-SKL into early-fertilized embryos. Histological sections taken of developmental stage 10 embryos revealed that peroxisomes were not visible, as GFP-SKL revealed a diffuse staining pattern that lacked punctate structures (Figure 8A), similar to that seen in control GFP injections embryos (data not shown). However, GFP-containing punctate bodies, indicative of peroxisomes, are readily visible in control embryos at stage 20 in the somites (Figure 8B).

Next, we wanted to determine if overexpression of Pex11β could induce an accumulation of peroxisomes, similar to how overexpression in A6 cells increased peroxisome numbers. Histological sections showed that ectopic expression of HA-Pex11β in embryos resulted in the presence of punctate GFP-containing bodies at stage 10 (Figure 8C), versus the diffuse pattern of GFP-SKL seen at this stage in the dorsal structures of uninjected embryos (Figure 8A). Further, there was also a relative increase in the number of punctate structures at stage 20 following HA-Pex11β and GFP-SKL (Figure 8D), compared to the microinjection of GFP-SKL alone (Figure 8B).

## Discussion

Pex11 proteins were first identified in yeast as peroxisomal membrane proteins that could increase peroxisome number when overexpressed and significantly reduce peroxisome number when interrupted [24]. Early studies suggested that Pex11 proteins acted primarily on medium-chain fatty acid oxidation, affecting peroxisome divisions indirectly [16]. Schrader and colleagues were the first to show in human fibroblasts that overexpression of human Pex11β was sufficient to induce peroxisome proliferation [25]. Recently, it has been shown that Pex11β participates in peroxisome divisions through membrane elongation and shape changes of existing peroxisomes. Elongated membranes fill with imported matrix proteins, form into small blebs and separate into new peroxisomes with the aid of dynamin-like protein [26]. While yeast studies have shown that peroxisomes only arise through division [27], and mammalian cell studies have suggested that they arise from both *de novo *and division mechanisms [28], little is known about peroxisome biogenesis during embryonic development. The question of peroxisome inheritance remains largely unresolved, particularly as we have shown that peroxisomes are absent in early frog embryos, and arise only later due to embryonic and or metabolic cues [29].

We tested whether overexpression of Pex11β could induce an early-onset to peroxisome biogenesis or accumulation during early Xenopus embryogenesis. This is particularly intriguing, as stage 10 embryos have no detectable peroxisomes. Thus, as Pex11β participates in peroxisome division, and no detectable peroxisomes are present in early embryos, Xenopus represents a novel model where the role of Pex11β in peroxisome number can be examined. The utility of microinjection and relative ease of expression and localization assays enables specific questions related to Pex11β to be addressed. First, we sought to show that Pex11β is sufficient to regulate peroxisome related protein and RNA levels, and increase the number of peroxisomes in *X. laevis *A6 cells. Our RT-PCR analysis indicated significant increases in RNA levels for both catalase and PMP70, amongst other genes, following overexpression of Pex11β. Using Western blot analysis we confirmed that HA-Pex11β increased catalase and PMP70 proteins levels, and immunohistochemistry confirmed that HA-Pex11β increased the number of both catalase and PMP70 positive punctate structures in A6 cells. Additionally, as GFP-SKL can be transported into peroxisomes, co-transfection of HA-Pex11β and GFP-SKL revealed an increase in the number of peroxisome-like structures. These results strongly support the idea that Pex11β can independently promote increases to the number of peroxisomes in Xenopus A6 cells.

The primary focus of our study was to elucidate the role of Pex11β *in vivo*. Very little is known about what cellular mechanisms regulate the *de novo *biogenesis of peroxisome during Xenopus development. Using a different GFP-KANL reporter, we had previously reported their detection at stage 30 in the ectoderm [29]. Histochemical studies in frog have suggested that yolk protein and lipid metabolism occurs at different stages in different tissues [22,30]. Interestingly, early yolk metabolism is seen in the newly formed muscles - the somites, but not in the large yolk-filled endodermal cells that are present on the ventral side of the embryo [22,30]. Here, using HA-Pex11β and other specific assays, we demonstrate that peroxisomes are detectable in somites at stage 20, but not at stage 10.

In agreement with the presence of peroxisome by stage 20, the RNA levels of most peroxisomal genes examined changed temporally during early development. Pex11β Pex3, Catalase, and PMP70 showed increasing trends in expression as development proceeded, peaking stage 30, with cytosolic-bound peroxisomal receptor Pex5 not varying during these stages. This suggested that transcripts are present and increasing towards the eventual onset of peroxisome biogenesis and/or their subsequent proliferation. These changes in Pex3 and Pex11β RNA levels relate well with previous studies that have demonstrated their roles in division [31]. If Pex11β did play a key regulatory role, we next determined how microinjecting HA-Pex11β mRNA would affect the relative levels of key peroxisomal genes. Changes of Pex11β RNA levels simply reflect and confirm the presence on the transfected construct. The Pex11β resulted in the significant increases in RNA levels for catalase and PMP70 at all stages tested (10, 20 and 30). There were also increases in the levels of Pex3 and Pex5 at two of the three stages examined, however, these changes were not as dramatic. From this data, we conclude that Pex11β can play a role in the early induction of these peroxisomal genes. Interestingly, as was examined with Pex11β in A6 cells, PPARα RNA levels increased, PPARγ decreased, and PPARδ was unchanged by ectopic Pex11β in embryos. Given that PPARα has roles in the β-oxidation of fatty acids, PPARγ role in lipid catabolism and adipocyte differentiation, and that while expressed ubiquitously, PPARδ functions remain unclear, the significance of our findings are not known. Furthermore, the relationship between PPARs, other metabolic regulators, yolk utilization and peroxisome numbers certainly bears further investigation.

We focused on the distribution of catalase and PMP70 protein within the somites and found that catalase and PMP70 proteins are first localized as punctate structures suggestive of peroxisomes at stage 20, with no detectable signal at stage 10. To corroborate this immunological finding we microinjected GFP-SKL RNA, whose product could be transported into peroxisomes. Our stage 10 histology sections revealed diffuse signals from GFP, indicating that peroxisomes are not yet present, as the SKL-tagged GFP was not localized. However, we were able to show that GFP-SKL localized to punctate-like structures in the somites at stage 20, indicating that peroxisomes are present at this stage.

With these results in mind, we next tested whether microinjecting HA-Pex11β RNA could induce an early accumulation to the number of peroxisomes. While peroxisomes are present at stage 20, perhaps all needed precursors are present earlier in the embryo and waiting a developmental or metabolic cue to form functional peroxisomes. Following the microinjection of HA-Pex11β, we were able to visualize peroxisome-like structures using GFP-SKL at stage 10. This suggested that needed peroxisomal precursors, including matrix proteins and other division proteins, such as dynamin-like proteins are present. Interestingly, together with the data that showed that HA-Pex11β injections increased the transcription of peroxisomal genes, this suggests that Pex11β is a key regulator of peroxisome onset and proliferation during Xenopus development. For the first time, we are able to show that Pex11β can independently induce an early onset to peroxisome accumulation *in vivo*.

## Conclusions

From our data we conclude that Xenopus Pex11β plays a role in regulating peroxisome number both in A6 cells *in vitro *and *in vivo *in embryos. Ectopic expression *in vivo *demonstrated for the first time Pex11β's ability to induce peroxisome related gene expression, and additionally to promote the early formation of peroxisome-like structures in embryos.

## Methods

### Animal Care

Adult *X. laevis *were reared in accordance with Canadian Council on Animal Care regulations. Fertilizations were performed according to Wu and Gerhart [32], and embryos were staged according to Nieuwkoop and Faber [33]. Embryos to be sectioned were fixed in 4% formaldehyde at desired stages and paraffin-embedded.

### Cloning, RNA Synthesis, and Microinjection

We cloned Xenopus full length Pex11β [GenBank:MGC69071] from total adult liver cDNA using specific primers using and SuperScript™ Reverse Transcriptase (Invitrogen) with Platinum^® ^*Taq *DNA Polymerase High Fidelity (Invitrogen) using conditions supplied by the manufacturer. A 5' HA tag was added to Pex11β using specific primers; HA-Pex11β 5'AGA TCT TCA AGC GTA ATC TGG TAC GTC GTA TGG GTA GGG CTT CAG CTT CAG CCA 3' and 5' CGA ACC CAC GAG TCC ATA CTA GT 3'. We also engineered GFP tagged with the PTS1 SKL, using forward 5' AGA TCT ATG GTG AGC AAG GGC GAG 3' and 5' ACT AGT CTA TAA TTT GGA CTT GTA CAG CTC GTC CA 3'. PCR products were cloned into the pCR^®^II-TOPO vector as per manufacturer's instructions (Invitrogen). Recombinant sequences were confirmed at the Robarts Research Institute DNA Sequencing Facility at the University of Western Ontario. Desired clones were additionally cloned into pcDNA™TOPO 3.3^® ^TA Cloning Kit (Invitrogen) for cell culture experiments, and T7TS plasmid and sequenced *in vitro *RNA production. Capped polyadenylated RNA was synthesized using mMachine mMessage^® ^T7 (Ambion) and visualized on a 1.0% agarose formaldehyde gel to ensure quality and transcription validity. Embryos at the one-cell stage in 4% ficoll in 1X Marks Modified Ringer (MMR) solution were microinjected with approximately 1 ng of desired RNA. Following 4 hours, embryos were transferred to 0.1X MMR for rearing.

### Cell lines, Transfections and Immunocytochemistry

A6 cells derived from *X. laevis *epithelial cells (generous gift from Dr. John Heikkila, University of Waterloo, ON) were grown in Leibowitz-15 media (with 10% FBS and 1% penicillin and streptomycin) at room temperature. All transfections were completed using Lipofectamine Plus LTX Transfection Reagents (Invitrogen) according to manufactures protocol. For immunofluorescence, cells were fixed in 3.7% formaldehyde in Dulbecco's modified PBS (DPBS) (Invitrogen), pH 7.4, for 10 min, and permeabilized in 1% Triton X-100 in DPBS for 10 min. Cells were incubated with either PMP70 (Abcam, ab4965) or catalase (Cedarlane), and/or haemagglutinin (Invitrogen) polyclonal antibodies for 3 hours, washed three times in PBST for 5 minutes each, incubated with fluorescently labeled secondary antibodies for 1 hour, washed again for 5 minutes in DPBS, and mounted on slides using ProGold mounting media (Invitrogen). Samples were visualized with a Zeiss AxioStop 2 Mot. Images were captured with a Retiga 1600 camera (Qimaging) and fluorescence quantifications were completed using Northern Eclipse image capture and analysis software (Empix).

### RNA Isolations and Reverse Transcriptase PCR (RT-PCR)

RNA was isolated from cell lysates of all samples two days following transfections. Total RNA was isolated with an RNeasy kit (QIAGEN) from embryos at developmental stages 10, 20, 30, and from A6 cells, was evaluated on a 1.0% agarose formaldehyde gel. Synthesis of cDNA was completed with SuperScript II Reverse Transcriptase (Invitrogen) following manufactures protocol. To analyze RNA expression levels during development, RT-PCR primers were designed against known Xenopus peroxisomal and PPAR as follows; Pex1 [NM_001091972.1] forward 5' CTT ATG GAG AAA TGT TTG GTT AAG A 3' and reverse 5' TTC ACG TGA TTG CAT TCT CAG ACT 3', Pex3 [EMBL:AAH73069.1] forward 5' ATC CAG CAT TCA GCA CCT TCT AGG 3' and reverse 5' ATC TGA TTC CTC CCC ATT TAG GC 3', Pex5 [NP_001011381] forward 5' CAG AAC AGG CAG ATC CCA TGT CCT 3' and reverse 5' ATC CTC TTT ACA AAC TGT AAG AAC 3', Pex11β [MGC69071] forward 5' TGC GAC AAC ATT CTG TGG GTC GGG AA 3' and reverse 5' CGA CAC CAT TAT CCT CAC TCA GT 3', PMP70 [EF07060] forward 5' TTG GAT GAT TCA GAA TGG TAC TT 3' and reverse 5' AAC CTT TCA ACA TCT TGT GTA AGC A 3', Catalase [BC054964] forward 5' GAG AAC ATT GGG AAG AGA ACT CCA AT 3' and reverse 5' CTT CAA ATG AGT CTG TGG GTT CCT TTT C 3', PPAR alpha [NM_001095362] forward 5' CCA TCC TGA TTG GGA AAG CCA GCA CTC 3' and reverse 5' CTA CGA GGC CAT GTT TGC CAT GCT GGC GT 3', PPAR delta [NM_001087841] forward 5' TTA CAG GAA CAG AGA TTG GAG TTC A 3' and reverse 5' CTC CAA GTT CAA TGC CCT GGA ACT TAA TG 3', PPAR gamma [NM_001087843] forward 5' AGG AGA AAT TAT TGG CTG AAA TCT CCA G 3' and reverse 5' GAC CTG AAC GAC CAA GTG ACG CTG CTG AA 3' and EF1-alpha forward 5' CAG ATT GGT GCT GGA TAT GC 3' and reverse 5' ACT GCC TTG ATG ACT CCT AG 3', genes with the following accession numbers: Pex3 [EMBL:AAH73069.1], Pex5 [NP_001011381], Pex11β [GenBank:MGC69071], PMP70 [EF07060], catalase [BC054964], Pex1 [NM_001091972.1], PPARα [NM_001095362], PPARδ [NM_001087841], and PPARγ [NM_001087843]. Mid-log phase RT-PCR products were visualized on a 1% agarose gel and unsaturated band intensities were quantified against control elongation factor-1α [NCBI: NM_001087442] with Quantity One software (Version 4.4.0 Bio-Rad). As the Xenopus genome has not been fully annotated, only primers for Pex3, PMP70 and EF1-α could be confirmed to span intron sequences. These particular primers sets therefore confirmed that there was no genomic DNA contamination in the cDNA samples used. All quantified PCR reactions were completed in triplicate. The amplicons of peroxisomal genes listed above were cloned with the TOPO-TA Cloning^® ^(Invitrogen) system as described by the manufacturer's protocol and sequenced to ensure gene identities.

### Western Blot Analysis

PMP70 (Abcam, ab4965), catalase (Cedarlane), hemagglutinin and β-actin (Invitrogen) polyclonal antibodies were used to detect protein from both *X. laevis *A6 cell lysates before and after treatments. Bradford protein quantifications were used to ensure that equivalent amounts of protein (10 mg) were loaded for each sample [34]. Primary antibodies were used in a 1 in 1000 dilution and secondary 1 in 10,000 dilution, and blots were developed using an enhanced chemiluminescence kit (Amersham). Band intensities were quantified using Quantity One software (Version 4.4.0 Bio-Rad).

### Immunohistochemistry

Paraffin-embedded embryo sections were washed in Xylene and re-hydrated by washing in 100, 90, 80 and 70% ethanol each for 10 minutes twice, followed by 10 min in 0.1% Tween-20 in PBS twice. Histology sections to be immunostained were incubated with a 24-hour primary followed by a two-hour secondary (FITC or Texas Red conjugated) antibody incubation in a 1 in 100 antibody dilution. Embryos were counterstained with DAPI (Invitrogen) according to the manufacturer's protocol. Images were captured and fluorescence quantified with a Zeiss LSM Dou (Live 5 Vario II and 510 Meta) Confocal system using Northern Eclipse image capture and analysis software (Empix).

### Statistical Analyses

Tests of significance are described within figure legends as required.

## Authors' contributions

MF designed and performed all experiments. MF and SD wrote, critically read and approved the final manuscript. MN helped with immunoblot interpretation. LW assisted with immunohistochemical experiments. All authors read and approved the final manuscript.
